# Magnetic Resonance Imaging Role in the Differentiation Between Atypical Cartilaginous Tumors and High-Grade Chondrosarcoma: An Updated Systematic Review

**DOI:** 10.7759/cureus.11237

**Published:** 2020-10-29

**Authors:** Salah M Alhumaid, Alwaleed Alharbi, Hamad Aljubair

**Affiliations:** 1 Department of Diagnostic Radiology, Prince Sultan Military Medical City, Riyadh, SAU

**Keywords:** mri imaging, chondroid tumors, chondrosarcoma, high-grade chondrosarcoma, atypical cartilaginous, tumors

## Abstract

Chondrosarcoma (CS) is a malignant tumor affecting the bones while atypical cartilaginous tumors (ACTs) are chondral tumors with moderate cellularity, mild atypia, and with myxoid changes and mild metastatic ability. Both can have one of the worst prognoses if not identified early enough. Magnetic resonance imaging (MRI) has been one of the modalities to detect such tumors and we aim to determine the common characteristic and features to be seen while screening for CS or ACTs. We conducted a systematic review of the previously published reports that investigated the diagnostic ability of MRI and the reported characteristics that can differentiate between ACTs and high-grade chondrosarcomas (HGCS). A comprehensive relevant database search was performed to include all the relevant studies. Among these studies, seven studies investigated the overall accuracy in the classification of the different chondroma types. Additionally, many studies reported the characteristic findings of each tumor according to the MRI results. These characteristics mainly included trapped fat, bone marrow edema, cortical damage, and soft-tissue expansion. Therefore, further attention should be given to these criteria for better assessment, differentiation, and favorable outcomes. MRI can efficiently identify some of the characteristics of both ACTs and HGCS. However, combining it with other radiological modalities may lead to a better differentiation. The detection of ACTs and HGCS lesions with MRI solely has been doubted before in the literature.

## Introduction and background

Chondrosarcoma (CS) is a malignant tumor that primarily affects bones. It is one of the most common tumors in this field following osteosarcoma and multiple myelomas and was first described in 1939 by Lichtenstein and Jaffe [1,2]. The main feature of this tumor is that it has a high tendency to produce cartilages where it commonly occurs in long bones as a central CS form. Furthermore, the histological nature of this tumor shows that it can be classified into three grades based on the cell; mitosis, cellularity, and atypia [3]. The three grades from one to three are classified according to their degree of malignancy as low, intermediate, and high. The incidence of CS is one per 200,000 per year with a rate of 30% for chondrosarcoma grade one (CS1), which is considered a non-metastatic low-grade tumor, but a locally aggressive one [4,5]. Although being local, this form of CS is usually treated by surgical removal followed by palliative radiotherapy as the biopsy is considered hazardous due to the high index of wrong sampling [6]. However, other non-surgical approaches have been introduced as successful alternatives. These include intralesional curettage together with the application of local adjuvant therapy [7,8]. Cartilaginous lesions of unknown malignant potential have been commonly used to describe atypical cartilaginous tumors grade 1 (ACTs1), and are usually used to describe chondral tumors with moderate cellularity, mild atypia, myxoid changes, and mild metastatic ability [9].

Overall, CS possesses a poor prognosis in general due to the potential ability to fail locally [10]. Furthermore, another form that is usually associated with a low-grade form is the dedifferentiated CS, which is a highly malignant sarcoma with no cartilage formation [11-14]. The other two grades of CS can metastasize as it has been previously reported in up to 30% of grade two (intermediate form) while it can reach up to 70% in grade three (highly malignant form) [15]. The differentiation between the grades of CS is of high importance due to the different prognosis of each grade, and therefore, each grade will have a specific therapeutic and follow-up approach [16-18]. The therapeutic approaches for these tumors, unlike CS1 or ACTs, are amputation or endoprosthetic reconstruction together with the application of the limb salvage technique [19].

Recently, the incidence of this tumor has rapidly increased and is usually discovered accidentally due to the high frequency of performing magnetic resonance imaging (MRI) in patients having bone or joint-related problems [20,21]. Therefore, MRI utilization is considered better for the detection and differentiation of such tumors. However, the diagnosis of this tumor is made on a bifactorial level by clinical findings as well as the results of the widely used imaging approaches. The majority of investigations reporting the efficacy of MRIs to differentiate such tumors have also focused on differentiating enchondroma (EC), which is the benign form of cartilaginous tumors, and grade one CS [22-24]. Moreover, identification of the different characteristics of ACTs on MRIs can be challenging as many studies have demonstrated many characteristics. Therefore, in this study, we aim to systematically review the efficiency of MRIs similar to what Deckers et al. [25] did previously but in a more systematic and characteristic approach, as well as added additional studies and content that was not included in that study, to differentiate between the different types of CS and identify the imaging criteria found in such imaging technique.

## Review

Search strategy, data collection, and study selection

Following a predefined protocol, collection of the relevant studies was performed through the Preferred Reporting Items for Systematic Reviews and Meta-Analyses (PRISMA) statement and guidelines [26]. Our criteria included many studies that investigated the efficacy of MRIs to differentiate between the different types of CS especially between low-grade and other advanced highly malignant forms of bone CS. For that, nine databases were searched including PubMed, metaRegister of Controlled Trials (mRCT), Scopus, Google Scholar, WHO Virtual Health Library (VHL), Web of Science (WoS), OpenGrey, Cochrane Library, and EMBASE.

The search strategy was tailored for each database based on three main elements: pathology/histopathology, MRI, and CS. Moreover, the used search term for PubMed was; “Histology” [MeSH],OR Histology [tiab],OR Histological [tiab],OR histopathology* [tiab],OR “pathology”[MeSH Terms] OR pathology[tiab] OR pathological[tiab])) AND ((“Magnetic Resonance Imaging”[MeSH] OR imaging[tiab] OR diagnostic imaging [Subheading] OR mri[tiab] OR DWI[tiab] OR MR scan*[tiab])) AND ((“Chondrosarcoma”[MeSH] OR Chondrosarcoma*[tiab] OR (cartilag*[tiab] AND (tumor*[tiab] OR tumour*[tiab] OR sarcom*[tiab]))). For databases not supporting medical subject heading (MeSH) terms, all possible word combinations were used to cover the possible terminologies. After performing the search strategy, all of the relevant results were exported to one endnote library for the detection and removal of the possible duplicates.

Afterward, all the remaining articles were exported to a standardized excel sheet for future methodology to include the relevant studies. At first, we performed title and abstract screening for a preliminary exclusion of irrelevant studies. Then, full-text download and screening were done to include all the relevant studies that met our criteria. These criteria included studies that investigated the diagnostic ability of MRI in the classification of the different forms of CS before biopsy or preoperatively based on the histopathological findings of these forms and the presence of reported characteristics of the imaging ability in studies that investigated adult patients. All of the screening was done independently by each author and any conflict was resolved by a proper discussion between the three parties to reach a final agreement.

Data extraction and risk of bias

After reaching the last decision on the final list of the included studies, data were prepared to go through the final step of data extraction to find the relevant outcomes according to the aim of the study. For this purpose, a sample of the included studies was used to construct a standardized extraction sheet that included all of the relevant outcomes including a sheet for the baseline characteristics as the last name of the first author, study year, country, sample size, and age of the included patients. Furthermore, a sheet for the pre-planned included outcome effectiveness of MRIs to differentiate between the different forms of CSs and the characteristics of this imaging. Another sheet was also established to assess the quality of the included studies. The final sheet held all the data extracted which was performed for all the included studies to fit all the relevant information. All of the authors who participated in the data extraction process were blinded from the results of their mates until finishing extraction, which was then followed by a public discussion to reach a final consensus about all of the extracted items. As a part of the extracted data, quality assessment elements were also extracted in the same way as mentioned above. For the assessment of bias, we used the risk of bias in non-randomized studies of interventions (ROBINS-I) tool [27] using the criteria that fit the design of our included studies.

Search results and study characteristics

**Figure 1:**
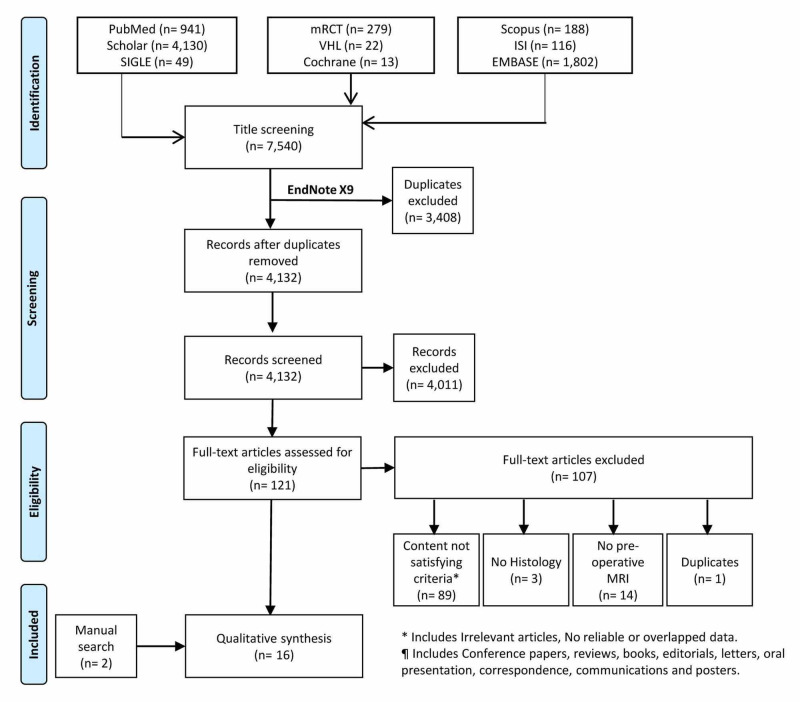
PRISMA flowchart of the search and screening process

Following the whole search process, we retrieved 7,540 records, out of those, 3,408 were duplicates, which left 4,132 studies to be screened. The title and abstract screening phase resulted in 121 papers for full-text screening, of which only 14 were retained. After adding two other studies that were searched manually, we finally included 16 studies for qualitative synthesis (Figure 1).

All the included studies were retrospective published between 2003 and 2020. Moreover, the total sample size was 568 patients with a range of 9 to 179 sample sizes. Among these studies, five studies were conducted in the United Kingdom [28-32], two in the United States [33,34], two in Germany [35,36], two in Italy [37,38], two in China [39,40], one in Korea [41], one in Japan [42], and one in Switzerland [43]. The baseline and summary of the included studies are presented in Table 1.

**Table 1:** Baseline characteristics of the included studies ACT: atypical cartilaginous tumors, ADC: apparent diffusion coefficient, CS: chondrosarcoma, DCE: dynamic contrast-enhanced, DWI: diffusion-weighted imaging, EC: enchondroma, HGCS: high-grade chondrosarcomas, HG: high grade, LG: low Grade, MRI: magnetic resonance imaging.

Study Reference	Study Year	Country	Design	Sample Size	Study Groups		MRI		Author Conclusion	Assessed Characteristics
					Groups	N	Type	Strength		
Crim et al. [33]	2015	United States	Retrospective	53	EC and SC	32/12	Conventional	NS	MRI can detect CS but with limitations.	Cortical breakthrough, soft-tissue expansion, scalloping, solid enhancement.
Douis et al. [30]	2014	United Kingdom	Retrospective	179	ACT/CS1/CS2/CS3	28/79/36/13/23	Conventional	NS	MRI can be used to differentiate between CS types by bone expansion, active periostitis, soft-tissue mass, and tumor length	Bone marrow edema, cortical breakthrough, soft-tissue expansion, bone expansion, soft-tissue edema, and periosteal edema.
Douis et al. [28]	2015	United Kingdom	Retrospective	52	ACT/CS1/CS2/CS3	5/15/3/2/3	DWI	3T	DWI cannot differentiate between CS types.	Cortical thickening, ADC.
Douis et al. [29]	2017	United Kingdom	Retrospective	60	CS1/CS2/CS3	15/3/1/4	Conventional	3T	Scalloping is the most sensitive MRI characteristics.	Entrapped fat, bone marrow edema, cortical breakthrough, soft-tissue expansion, bone expansion, scalloping, soft-tissue edema, and periosteal edema.
					CS1/CS2/CS3	15/3/1/4	DCE	3T	DCE is not useful in differentiating.	DCE MRI parameters.
Errani et al. [37]	2017	Italy	Retrospective	89	EC/ACT	54/35	Conventional	1.5T	Scalloping and soft-tissue extension of the most sensitive MRI characteristics.	Soft-tissue expansion, scalloping.
Fayad et al. [34]	2015	USA	Retrospective	24	CS2/CS3	1-Jun	Conventional	1.5T	Scalloping and soft-tissue extension of the most sensitive MRI characteristics.	Soft-tissue expansion, solid enhancement, soft-tissue edema, and periosteal edema.
Gitto et al. [38]	2020	Italy	Retrospective	58	ACT/HGCS	26/38	Conventional	1.5T	Machine approaches can easily classify CS types.	
Kang et al. [39]	2016	China	Retrospective	31	CS1/HGCS	15-Jun	Conventional	1.5T	Cortical destruction, hip joint infiltration and tumor size, soft-tissue mass is useful in differentiation.	Bone marrow edema, cortical breakthrough, soft-tissue expansion, and lobular outer margin
Lisson et al. [35]	2018	Germany	Retrospective	22	EC	11-Nov	3D texture analysis	1.5 or 3T	MRI-based 3D texture analysis can differentiate between EC and CS.	
Liu et al. [40]	2017	China	Retrospective	23	17 CS dd cases	-	Conventional	3T	No favorable outcomes.	Soft-tissue expansion, arc enhancement, arc enhancement, and Internal lobular architecture.
MacSweeney et al. [31]	2003	United Kingdom	Retrospective	9	8 CS dd cases	-	Conventional	1 or 1.5T	T2-weighted or STIR MR sequences can identify areas of dedifferentiation, which should be the preferential site of pre-operative biopsy.	Soft-tissue expansion
Muller et al. [43]	2016	Switzerland	Retrospective	96	EC/CS	8-Nov	DWI	NS	DWI can differentiate between CS types.	ADC
Saifuddin et al. [32]	2020	United Kingdom	Retrospective	52	-	-	T2-weighted fast spin-echo	1.5T	MRI can be used to differentiate between CS types.	Bone marrow edema, cortical breakthrough, soft-tissue expansion, bone expansion, soft-tissue edema, and periosteal edema.
Welzel et al. [36]	2018	Germany	Retrospective	105	ACT/HGCS	39/14	DWI	3T	Mean ADC value has higher accuracy rates.	ADC
Yoo et al. [41]	2009	Korea	Retrospective	42	LG/HG	28/14	Conventional	1 or 1.5T	Soft-tissue mass formation favored the diagnosis of HGCS, and entrapped fat within the tumor was highly indicative of LGCS.	Entrapped fat, soft-tissue expansion, soft-tissue expansion, arc enhancement, Internal lobular architecture, lobular outer margin, central non-enhancement region, solid enhancement.
Yoshimura et al. [42]	2013	Japan	Retrospective	17	CS1/CS2/CS3	6/10/2001	Conventional	NS	No favorable outcomes.	Entrapped fat, soft-tissue expansion, soft-tissue expansion, arc enhancement, Internal lobular architecture, and central non-enhancement region.

Quality assessment results

The overall quality of the included study varied hugely. According to the risk-assessment results, we found that seven studies [30-32,34,39-41] had a low risk of bias while only two were classified as high-risk studies [29,36]. In terms of confounding bias, only two studies [34,42] had a moderate risk of bias while none of the studies had a high risk. Missing data were found to cause a high risk of bias in two of the included studies [31,33] (Appendices).

Accuracy of MRIs in diagnosis and detecting characteristics

Among all the included studies, only seven studies [29,30,32,33,35,36,42] reported the efficacy and overall accuracy in the diagnosis and differentiation of CS. Saifuddin et al. reported an overall accuracy of 92% in a pre-biopsy-two-reader approach with a sensitivity of 91% [32]. Meanwhile, Yoshimura et al. reported high sensitivity rates in terms of entrapped fat, ring and arc enhancement, and soft-tissue mass formation characteristics [42]. However, the authors found that computed tomography and plain radiography were more sensitive in the detection of calcification, scalloping, and cortical penetration. Therefore, the authors suggested the combined utilization of both modalities for better accuracy. Furthermore, Welzel et al. reported high sensitivity and specificity rates for MRI in terms of apparent diffusion coefficient (ADC) values between chondroma and CS [36]. Lisson et al. reported an overall accuracy of 86% and 82% for the detection of kurtosis and entropy that are essential for discrimination between chondroma and CS [35]. Similarly, Douis et al. reported a positive predictive value (PPV) of 100% for the detection of cortical destruction and soft tissue masses, and 70% only for bone expansion [29]. Douis et al. reported an overall accuracy of 95.6% for MRI diagnostic ability [30]. Lastly, Crim et al. reported a 14% false-positive rate of MRI in diagnosing malignancy, which was much higher than the rate reported with radiography (3.1%) [33].

MRI reported characteristics

Various MRI characteristics have been identified and are discussed as follows. Entrapped fat was reported by three of the included studies [29,41,42]. Among these studies, Yoo et al. and Douis et al. reported that the presence of entrapped fat or fatty pools was significantly observed in ACTs more than HGCS (P<0.01) [29,41]. In the same context, Yoshimura et al. found that the number of entrapped fat observations was more in the ACT group (26/28) than the HGCS one (1/14), with a reported PPV of 90% in this point [42].

Characteristic detection of edema

Regarding bone marrow edema, it was reported by only three studies [29,30,39]. Among these studies, Kang et al. reported that the presence of peritumoral edema was observed in all of the patients in the ACT and HGCS groups, and therefore, no significance was found [39]. In the same context, Douis et al. [30] in their study in 2014, reported statistical significance between the two groups while in their study that was conducted in 2018 [29], a statistical significance was found in the presence of bone marrow edema among all chondroma tumors. However, a higher rate was noticed among the HGCS patients (6/8) versus ACT patients (7/15). Similarly, the presence of soft-tissue and periosteal edema was compared between ACT and HGCS by two of the included studies [29,30]. Both studies found a significant relationship between the two groups; however, Douis et al. [29] results might have been influenced by the heterogeneity of the results between the different groups of chondromas that were included. Lastly, Fayad et al. reported a high rate in the presence of soft-tissue edema and periosteal edema; however, the authors included only patients with grade 2-3 CS with no comparison between them and ACT [34].

Characteristics detection of expansion

The bone expansion reported by Douis et al. in 2014 and 2015 reported that a higher rate of bone expansion was significantly associated with HGCS than ACT [28,30]. On the other hand, soft-tissue expansion was reported by 10 studies [29-31,33,34,37,39-42]. Among these, only five of them [29,30,33,37,39,41,42] compared the significance of the presence of the characteristic on MRIs while Crim et al. [33] and Errani et al. [37] reported pure rates only in ACT patients without comparing them with rates of 25% and 23.5%, respectively. Meanwhile, Fayad et al. [34], Macsweeney et al. [31], and Liu et al. [40] reported that almost all of their HGCS population were observed to have soft-tissue expansion on MRIs. Moreover, among the studies that compared ACT and HGCS, significantly higher rates were associated with the HGCS group in all of them with scalloping being reported as one of the characteristics. Nonetheless, Douis et al. [28], reported that all of their patients had developed scalloping. However, Crim et al. [33] and Errani et al. [37] found smaller rates in the ACT group (25% and 52.9%, respectively) with no reported outcomes regarding the HGCS.

Characteristics related to the cortex and overall architecture

The lobular internal architecture was significantly present in the ACT group according to Yoshimura et al. [42] and Yoo et al. [41]. In HGCS, two studies [40,42] reported a high rate of detection; however, Yoo et al. [41] reported the lowest rate, which was significant when compared to the ACT group (P<0.01). The lobular outer margin was observed in almost all of the patients in the ACT and HGCS groups [39,41]. However, Yoo et al. [41] found a lower rate in the HGCS group (71%) with an estimation associated differences in the incidence between the two groups (P<0.05). A breakthrough in the cortex was also present more significantly in the HGCS group than the ACT one as reported by four studies [29,30,39,41] compared to Crim et al. [33] where they reported a rate of 25% in their small ACT population with no comparison. In the same context, Yoo et al. [41] found that the destruction of the cortex was significantly observed in HGCS patients than with ACT (P<0.01). Additionally, Douis et al. [30] reported that none of the ACT patients had cortical thickening while a rate of 22% of the HGCS population developed these characteristics.

Characteristics related to enhancements

Regarding the comparison between the ring and arc enhancements between the ACT and HGCS groups. Yoo et al. outlined that 100% of his population's MRI results outlined enhancements while a significantly higher rate was observed in the ACT group than the HGCS where no enhancements were reported [41]. Another two studies [34,40] reported enhancements with the HGCS group reinforcing the results outlined by Yoo et al. [41]. However, the two studies did not compare their results to the HGCS group. Solid enhancement was also reported by three studies [33,34,41] with Yoo et al. study reporting a better enhancement finding in the HGCS group. Additionally, Fayad et al. [34] and Crim et al. [33] described moderate enhancement rates in the HGCS and ACT groups. Lastly, a central area of non-enhancement was significantly observed in the HGCS group than the ACT one [41,42].

Other reported outcomes

Assessment of tumor size was reported to be significantly larger in HGCS than ACT by Kang et al. [39] while Douis et al. [29] found no significant association. Most of the included studies [29-31,33,34,37,39-42] used the conventional MRI approach to assess their patients while another three studies [28,36,43] used the diffusion-weighted imaging (DWI) MRI approach which was mainly conducted to assess ADC. Moreover, Douis et al. [28] found no significance in ADC while Welzel et al. [36] reported that ADC was higher in grade 1 than grade 2 ACT, meanwhile, Müller et al. [43] reported high values with grade 1 but did not have any comparison. Lisson et al. [35] observed another two characteristics including kurtosis and entropy using contrast-enhanced and non-contrast T1-weighted MRI images by 3D analysis. However, the authors did not make a comparison between ACT and HGCS but did compare between EC and grade 1 CS. Douis et al. also used another approach and found no significance between the ACT and HGCS groups according to the dynamic contrast-enhanced (DCE) MRI findings [29]. Gitto et al. used an unenhanced MRI to compare the radiologist-based and machine-based evaluation of ACT and HGCS and showed that the latter could add to the diagnosis of both lesions, but with no statistical significance [38].

Limitations to our study include the relatively smaller sample size of the included studies. Moreover, we observed heterogeneity between the reported MRI characteristics of the included studies with no clear and defined outcomes between all of them. The definition of MRI accuracy was hugely variable among the studies which may have made it difficult for comparison.

## Conclusions

The overall reported accuracy of MRI as a differentiating tool between ACT and HGCS was generally acceptable and even high in some detected characteristics. However, it has been reported that the combined use of MRI and radiography will result in more accuracy. Additionally, the main characteristics that could be detected by MRIs include peritumor edema, entrapped fat, cortical damage, and expansion of the extra-osseous soft tissue. These can effectively differentiate between ACT and HGCS according to the included studies with further studies needed on a larger sample-sized population.

## Figures and Tables

**Figure 2:**
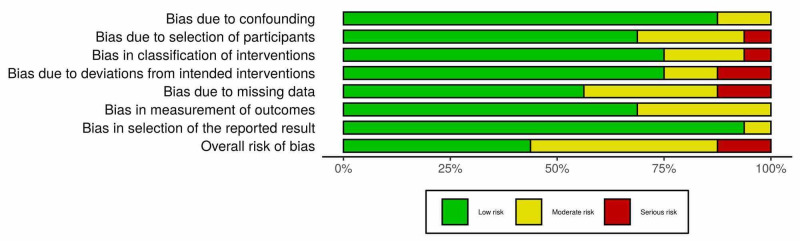
Risk of bias graph: review authors' judgments about each risk of bias item presented as percentages across all included studies

**Figure 3:**
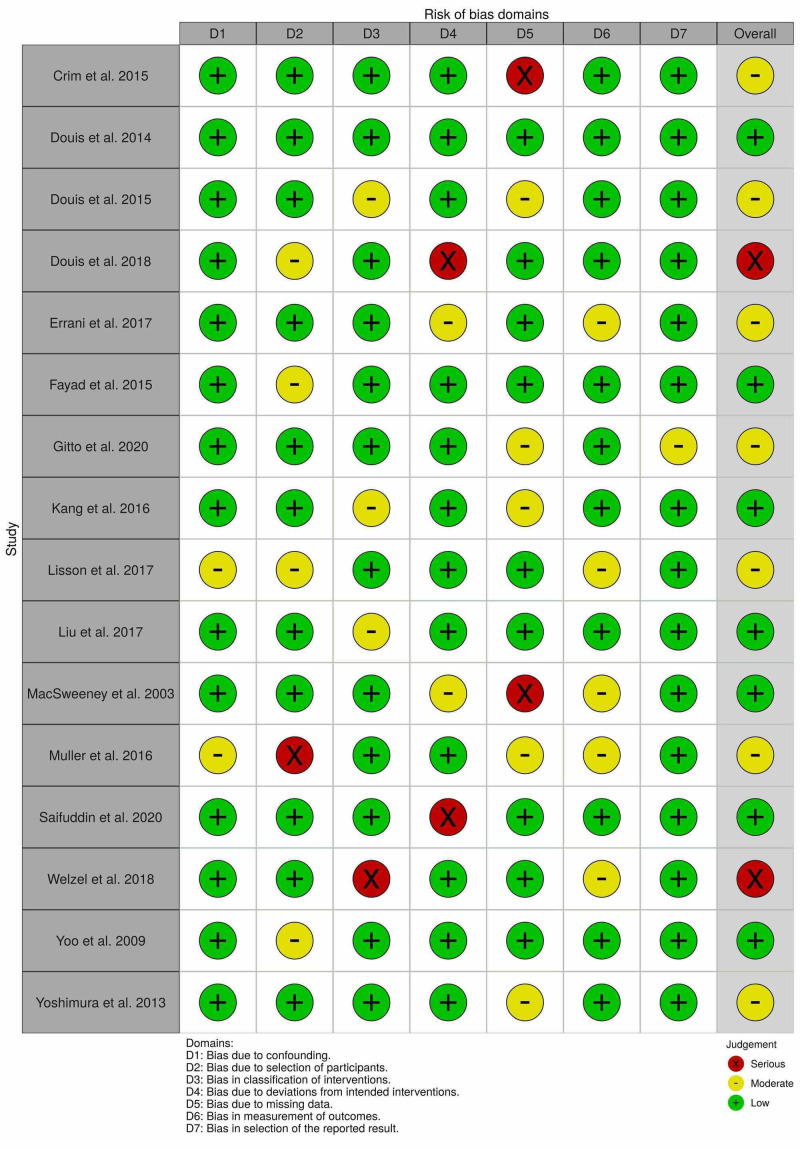
Risk of bias summary: review authors' judgments about each risk of bias item for each included study
